# Pharmacokinetics of High-Dose Weekly Oral Vitamin D3 Supplementation during the Third Trimester of Pregnancy in Dhaka, Bangladesh

**DOI:** 10.3390/nu5030788

**Published:** 2013-03-12

**Authors:** Daniel E. Roth, Abdullah Al Mahmud, Rubhana Raqib, Evana Akhtar, Robert E. Black, Abdullah H. Baqui

**Affiliations:** 1 Department of International Health, The Johns Hopkins Bloomberg School of Public Health, 615 North Wolfe Street, Baltimore, MD 21205, USA; E-Mails: rblack@jhsph.edu (R.E.B.); abaqui@jhsph.edu (A.H.B.); 2 International Center for Diarrhoeal Disease Research, Bangladesh (ICDDR,B), GPO Box 128, Dhaka 1000, Bangladesh; E-Mails: mahmud@icddrb.org (A.A.M.); rubhana@icddrb.org (R.R.); evana@icddrb.org (E.A.)

**Keywords:** vitamin D, Bangladesh, pregnancy, pharmacokinetics, hypercalcemia

## Abstract

A pharmacokinetic study was conducted to assess the biochemical dose-response and tolerability of high-dose prenatal vitamin D3 supplementation in Dhaka, Bangladesh (23°N). Pregnant women at 27–30 weeks gestation (*n* = 28) were randomized to 70,000 IU once + 35,000 IU/week vitamin D3 (group PH: pregnant, higher dose) or 14,000 IU/week vitamin D3 (PL: pregnant, lower dose) until delivery. A group of non-pregnant women (*n =* 16) was similarly administered 70,000 IU once + 35,000 IU/week for 10 weeks (NH: non-pregnant, higher-dose). Rise (∆) in serum 25-hydroxyvitamin D concentration ([25(OH)D]) above baseline was the primary pharmacokinetic outcome. Baseline mean [25(OH)D] were similar in PH and PL (35 nmol/L *vs.* 31 nmol/L, *p* = 0.34). A dose-response effect was observed: ∆[25(OH)D] at modeled steady-state was 19 nmol/L (95% CI, 1 to 37) higher in PH *vs.* PL (*p* = 0.044). ∆[25(OH)D] at modeled steady-state was lower in PH *versus* NH but the difference was not significant (−15 nmol/L, 95% CI −34 to 5; *p* = 0.13). In PH, 100% attained [25(OH)D] ≥ 50 nmol/L and 90% attained [25(OH)D] ≥ 80 nmol/L; in PL, 89% attained [25(OH)D] ≥ 50 nmol/L but 56% attained [25(OH)D] ≥ 80 nmol/L. Cord [25(OH)D] (*n* = 23) was slightly higher in PH *versus* PL (117 nmol/L *vs.* 98 nmol/L; *p* = 0.07). Vitamin D3 was well tolerated; there were no supplement-related serious adverse clinical events or hypercalcemia. In summary, a regimen of an initial dose of 70,000 IU and 35,000 IU/week vitamin D3 in the third trimester of pregnancy was non-hypercalcemic and attained [25(OH)D] ≥ 80 nmol/L in virtually all mothers and newborns. Further research is required to establish the safety of high-dose vitamin D3 in pregnancy and to determine if supplement-induced [25(OH)D] elevations lead to maternal-infant health benefits.

## 1. Introduction

The maternal-infant health benefits of vitamin D supplementation during pregnancy remain uncertain [1,2]. However, observational studies have suggested associations between vitamin D status during pregnancy and postnatal infant health outcomes [3,4,5]. Serum 25-hydroxyvitamin D concentration ([25(OH)D]) ≥ 50 nmol/L is associated with skeletal health benefits [1], but some data suggest that improving vitamin D status to attain serum [25(OH)D] ≥ 80 nmol/L may enhance a range of vitamin D-related functions [6,7,8]. However, there have been relatively few published studies of vitamin D3 pharmacokinetics, safety and clinical effects during pregnancy [9]. 

The possible association between maternal-fetal vitamin D status and infant health outcomes may be particularly relevant to South Asian countries such as Bangladesh, where adverse perinatal outcomes and infant mortality are public health priorities [10], and where vitamin D deficiency has been observed among women of reproductive age [11] and young infants [12]. Therefore, to guide the design of clinical trials of antenatal vitamin D supplementation in Bangladesh, we conducted a randomized open-label pilot trial of two antenatal vitamin D3 supplementation doses that were several fold higher than those in typical prenatal supplements. The primary aims were to establish the biochemical dose-response in terms of the change in serum [25(OH)D], and to specifically assess whether the regimens achieved [25(OH)D] ≥ 80 nmol/L in most participants. The response to the higher-dose supplement regimen was also assessed in a cohort of non-pregnant participants that served as a separate comparison group. The present study builds on previously reported observations of single-dose vitamin D3 pharmacokinetics in the same setting [13]. 

## 2. Experimental Section

### 2.1. Participants

Pregnant women were enrolled at a maternal health clinic in inner-city Dhaka, Bangladesh (23°N) in February 2010 if they were: Aged 18 to <35 years; at 27 to <31 completed weeks of gestation based on the reported first day of the last menstrual period; held permanent residence in Dhaka at a fixed address; and, planned to stay in Dhaka for at least four months. Reasons for exclusion were: preexisting medical condition; current vitamin D supplement use; anti-convulsant or anti-mycobacterial medications; severe anemia (hemoglobin concentration <70 g/L); hypertension at enrollment (systolic blood pressure ≥140 mmHg or diastolic blood pressure ≥90 mmHg on at least two measurements); major risk factors for preterm delivery or pregnancy complications; or previous delivery of an infant with a congenital anomaly or perinatal death. Healthy non-pregnant women attending the same clinic for health maintenance (e.g., contraception), or because they were accompanying pregnant women, were enrolled in August–September 2009 if they were non-lactating, had not missed a recent menses at the expected date, and had a negative urine pregnancy test (First Response Early Results, Church & Dwight Company, Inc., Princeton, NJ, USA). Otherwise, inclusion and exclusion criteria were similar to the pregnant participants.

The study was approved by the Institutional Review Board at The Johns Hopkins Bloomberg School of Public Health and the International Center for Diarrheal Disease Research, Bangladesh (ICDDR, B). All participants gave signed informed consent prior to participation. The trial was registered at ClinicalTrials.gov (NCT00938600).

### 2.2. Study Design and Interventions

Pregnant participants were randomized at enrollment to receive a single dose of vitamin D3 70,000 IU (1.75 mg, where 1 mg = 40,000 IU) on day 0 followed by vitamin D3 35,000 IU (0.875 mg) per week starting on day 7 and continuing until delivery (referred to as group “PH”; pregnant, higher dose), or to vitamin D3 14,000 IU (0.350 mg) per week starting on day 0 and continuing until delivery (“PL”; pregnant, lower dose). Participants in the non-pregnant cohort (“NH”; non-pregnant, higher dose) received the same higher-dose intervention as PH, *i.e.*, a single dose of vitamin D3 70,000 IU on day 0 followed by vitamin D3 35,000 IU per week starting on day 7 and continuing until the last dose on day 63 (total of 10 doses). Vitamin D3 was administered as Vigantol Oil (Merck KGaA, Germany), a liquid supplement (20,000 IU D3/mL) commercially available in Bangladesh (see Ref 13 for details regarding quality assurance). Participants were advised not to take other vitamin D-containing supplements during the study period. Pregnant participants were provided with standard prenatal supplemental iron (60 mg/day) and folic acid (400 mcg/day). NH was studied before enrolment of PH, to establish safety of the high-dose regimen in non-pregnant women prior to its use in pregnant women. As an additional safety measure, the response to a single initial dose vitamin D3 (70,000 IU) was observed in a separate cohort, prior to the initiation of enrollment of cohorts of participants who received weekly doses [13]. A preceding report of single-dose vitamin D3 pharmacokinetics included data from participants in weekly-dose groups PH and NH, but only from days 0 to 7 (*i.e.*, preceding the administration of a second vitamin D dose) [13]. Women who received only the single 70,000 IU dose are not included in any of the present analyses.

### 2.3. Data Collection Procedures

**Figure 1:**
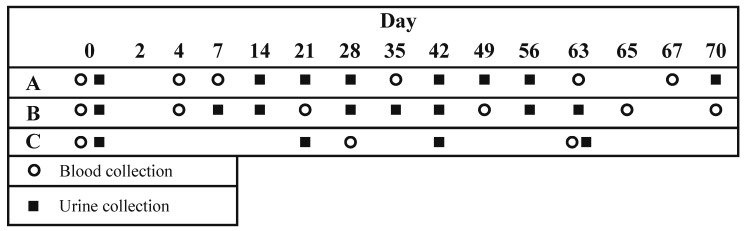
Blood and urine specimen collection schedules. Participants in groups PH and NH were randomly assigned to either scheduled “A” or “B”. Participants in group PL all followed schedule “C”.

Pregnant women were assessed weekly until delivery. Non-pregnant participants had weekly follow-ups for 10 weeks (the last visit was on day 70, one week after the final D3 dose). Weekly assessments included a checklist of symptoms and blood pressure measurement. In NH and PH, participants provided up to six scheduled blood specimens and at least seven urine samples during a 10-week follow-up period beginning on the day of supplement administration (day 0), according to one of two randomly assigned sampling schedules, A or B (Figure 1). Specimens on days 65 and 67 were intended to measure inter-dose fluctuations in [25(OH)D] and serum calcium. Urine was collected at visits without scheduled blood collection up to day 70 (Figure 1). Participants in PL were asked to provide three blood specimens and four urine specimens (schedule C in Figure 1). From day 70, pregnant participants provided urine specimens on a weekly basis until delivery. 

### 2.4. Specimen Collection and Biochemical Analyses

Maternal and cord serum samples were collected by standard techniques and maintained at 4 °C prior to same-day transfer to the laboratory. Spot urine specimens were collected in sterile plastic containers and maintained at 4 °C until same or next-day analysis of the calcium:creatinine ratio (ca:cr). Serum aliquots for the 25(OH)D assay were frozen at −20 °C for up to five months prior to shipment from Bangladesh to Toronto. Total serum [25(OH)D] was measured with the Diasorin Liaison Total assay in the laboratory of Reinhold Vieth in Toronto [14], which meets the International Vitamin D External Quality Assessment Scheme (DEQAS) performance targets [15]. Mean within-run coefficient of variation (CV%) was 7.8% (5.8% for specimens with values <150 nmol/L) and mean between-run CV% was 10.5% (9.0% for specimens <150 nmol/L). Serum calcium, serum albumin, and urine calcium:creatinine ratio (ca:cr) were routinely measured using the AU640 Olympus Autoanalyzer (Olympus Corporation, Japan) in the Clinical Biochemistry Laboratory at the International Center for Diarrheal Disease Research, Bangladesh (ICDDR, B) in Dhaka within 24–48 h of collection of serum or urine aliquots. Total serum calcium concentration ([Ca]) was adjusted for the serum albumin concentration by the following conventional formula: [Ca] + (0.02 × (40-albumin)). Intact parathyroid hormone (PTH) was measured using a chemiluminescent assay on the i1000SR Architect Autoanalyzer (Abbott Diagnostics, Lake Forest, IL, USA), with a reference range of 1.59–7.23 pmol/L (Clinical Biochemistry Lab, icddr,b).

### 2.5. Safety Monitoring

The adjusted [Ca] reference range was 2.10–2.60 mmol/L. Umbilical cord venous serum [Ca] was considered elevated if greater than 3.0 mmol/L [16]. Urine ca:cr were expressed as mmol Ca/mmol Cr, considering 1.0 as the upper limit of the reference range [17]. An albumin-adjusted serum calcium concentration >2.60 mmol/L prompted a repeat measurement on a new specimen as soon as possible. Confirmed hypercalcemia was *a priori* defined as albumin-adjusted serum calcium concentration >2.60 mmol/L on both specimens (since hypercalcemia caused by vitamin D intoxication would not be expected to resolve within a few days without intervention). Episodes of urinary calcium:creatinine ratio (ca:cr) >1.0 mmol/mmol prompted a repeat urine ca:cr measurement within one week. A ca:cr > 0.85 mmol/mmol that was also 2-fold or greater relative to the lowest previously observed value in the same participant prompted repeat urine assessment. Persistent hypercalciuria was defined as ca:cr > 1.0 mmol/mmol on two consecutive results, or on two non-consecutive measurements but in the presence of persistent symptoms suggestive of possible hypercalcemia. Persistent hypercalciuria or persistent ca:cr > 0.85 mmol/mmol that was also 2-fold or greater relative to the lowest previously observed value were indications for unscheduled measurement of serum calcium. Abnormal urinalyses, hypertension, reported severe symptoms, or persistence of any mild symptomatic complaints prompted referral to the study physician for further evaluation. Participants were referred to an antenatal care physician at the maternity clinic for treatment of urinary tract infections, hypertension, or other medical problems. Participants with obstetric complications were transported to a local tertiary-care hospital with advanced neonatal care facilities. All costs of medical and obstetric care were borne by the study. 

### 2.6. Statistical Analysis

Pharmacokinetic outcomes were expressed as the attained maternal/cord [25(OH)D] and the rise in maternal [25(OH)D] above baseline (Δ[25(OH)D]). Distributions in each group and at specific time points were summarized as geometric mean [25(OH)D] and 95% confidence intervals (CI). Between-group differences were analyzed by linear regression of log-transformed [25(OH)D]. To facilitate comparisons to other studies, the Δ[25(OH)D] at days 63 and beyond was also expressed as a function of the equivalent daily dose administered to each group, in micrograms (*i.e.*, 125 mcg/day in groups NH and PH, and 50 mcg/day in group PL). To investigate inter-dose fluctuations, the mean [25(OH)D] at days 65, 67, and 70 were compared to day 63 in groups NH and PH. The proportion of participants and cord blood specimens with [25(OH)D] ≥ 50 nmol/L or ≥80 nmol/L were compared across groups using log-binomial regression. Mean changes in [25(OH)D] over time in each group were also modeled as continuous non-linear parametric functions (see Appendix). These analyses used all available individual participant-level data; standard errors were corrected to account for the within-subject correlation of repeated outcomes. Serum [Ca] and urine log-transformed ca:cr were each modeled as functions of time using fixed indicator variables for baseline, weeks 2 to 5 (days 4 to 34), and week 6 and later (day 35 and thereafter). Comparisons of PH to NH or PL were analyzed using group-by-time interaction terms. Serum [Ca] and urine ca:cr were also expressed in terms of the proportions of episodes above the references ranges. In all analyses, *p* < 0.05 was considered statistically significant; however, the Holm procedure was used for multiple pair-wise comparisons [18]. Where appropriate, generalized estimating equations (GEE) with robust error estimation were used to account for non-independence of repeated measures. Analyses were conducted using Stata versions 10.1 and 11.1 (Stata Corporation, College Station, TX, USA).

## 3. Results

### 3.1. Participant Characteristics and Retention.

**Figure 2:**
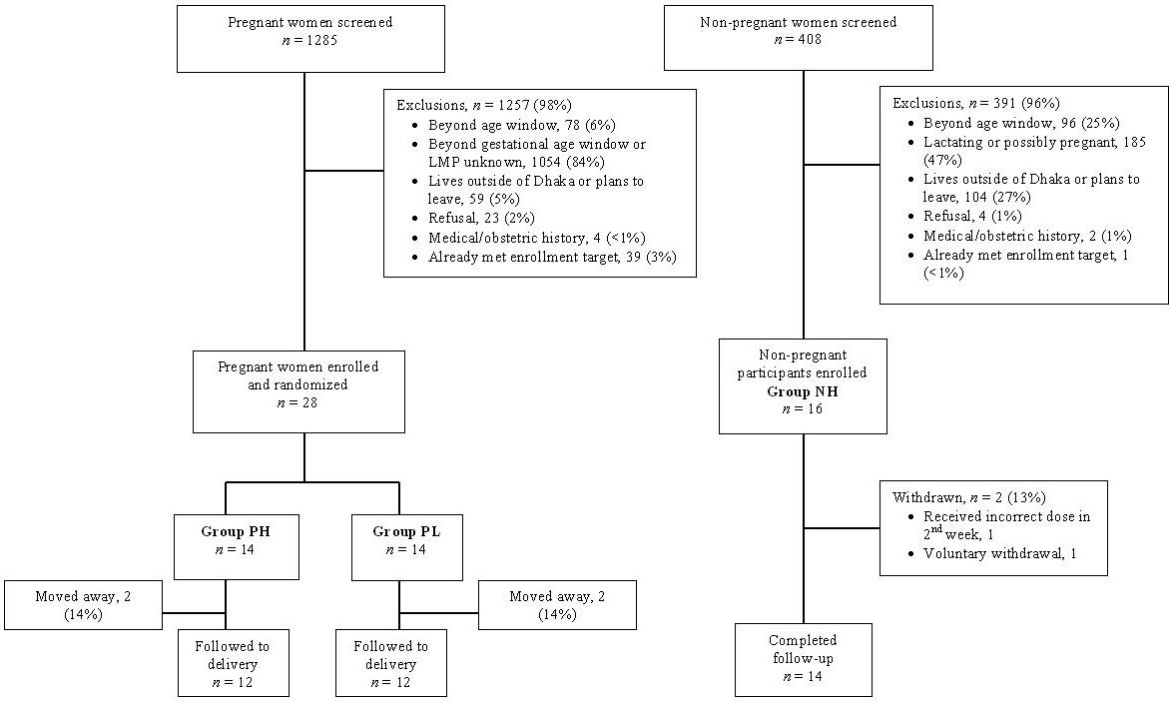
Study flow diagram. Participant screening, enrollment, exclusions, and withdrawal over the course of the study.

Twenty-eight pregnant women were recruited and randomly assigned to one of two groups, PH (*n* = 14) and PL (*n =* 14). Sixteen non-pregnant women were enrolled (Figure 2). 

Of 28 randomized pregnant participants, 4 (14%) left the Dhaka area prior to completion of the study protocol (2 in PH and 2 in PL). Specimens in the 10th week were available in 10/14 women in PH and 9/14 in PL. Cord specimens were available in 23 (82%) of enrolled participants. PH and PL were generally similar with respect to baseline characteristics (Table 1) and [25(OH)D] (Table 2). However, NH enrollment occurred in the summer rather than mid-winter and NH participants had higher average baseline [25(OH)D] compared to the pregnant participants (Table 1).

**Table 1:** Participant characteristics at enrollment.

		NH	PH	PL	*p*
**# Enrolled**		16	14	14	
**Age (years), Mean (±SD)**		24.6 (±4.5)	22.2 (±3.1)	22.1 (±4.8)	0.190
**Gestational age at enrollment (weeks)**					
	**Mean (±SD)**	-	28.4 (±1.2)	28.5 (±1.3)	0.760
	**Range**	-	26.1-30.6	27-30.7	
**Married**		12 (75%)	14 (100%)	13 (93%)	0.110
**Education level attained**					
	**None**	2 (13%)	4 (29%)	3 (21%)	0.285
	**Primary**	10 (63%)	9 (64%)	11 (79%)	
	**Secondary or higher**	4 (25%)	1 (7%)	0	
**Height (cm), mean (±SD)**		152.1 (±4.7)	150.7 (±4.7)	148.9 (±4.5)	0.179

**Table 2:** Serum 25-hydroxyvitamin D concentrations at baseline and through 10 weeks of supplementation in non-pregnant and pregnant participants^1^.

		Non-pregnant	Pregnant	Pregnant		
		NH	PH	PL		
**# Enrolled**		16	14	14		
**Vitamin D3 regimen**						
	**Loading dose**	70,000 IU	70,000 IU	0		
	**Weekly doses**	35,000 IU	35,000 IU	14,000 IU		
**Duration of supplementation**		10 weeks	27–30 weeks gestation until delivery	27–30 weeks gestation until delivery		
**Dates of enrollment**		17 Aug–6 Sep 2009	3–16 Feb 2010	3–16 Feb 2010		
**Participants with [25(OH)D] measured during 10th week (days 63 to 70), *n* (%)**		14 (88%)	10 (71%)	9 (64%)	***p* value** **^2^**	
**Number of specimens per participant,** ** Median **		6	6	3	**PH *vs.* NH**	**PH *vs.* PL**
**Baseline [25(OH)D]**						
	**Mean [95% CI]**	57 [47,69]	35 [30,42]	31 [26,38]	<0.001	0.341
	**Range (min, max)**	27, 93	21, 55	20, 57		
**Attained [25(OH)D] in 10th week**						
	**Mean [95% CI]**	139 [121,160]	98 [89,109]	76 [61,95]	<0.001	0.038
	**Range (min, max)**	85, 238	71, 153	36, 119		
**∆[25(OH)D] in 10th week**						
	**Mean [95% CI]**	76 [61,96]	57 [44,73]	36 [22,61]	0.082	0.128
	**Range on days 63 to 70**	28, 160	19, 130	7, 75		
**∆[25(OH)D] at days 63 to 70 per daily vitamin D3 dose (nmol/L/mcg)**						
	**Mean [95% CI]**	0.61 [0.48, 0.79]	0.46 [0.34,0.61]	0.73 [0.38,1.38]	0.220	0.081
**Area under the ∆[25(OH)D]-time curve (nmol·d/L) to day 63/65 (AUC_63_)** **^3^**		3500 [2886,4245]	2925 [2331,3670]	1678 [923,3053]	0.383	0.020
**Participants with mean [25(OH)D]**						
**≥ 50 nmol/L in 10th week, *#/n* (%)** **^4^**		14/14 (100%)	10/10 (100%)	8/9 (89%)	1.000	0.166
**Participants with mean [25(OH)D]**						
**≥ 80 nmol/L in 10th week, *#/n* (%)** **^4^**		14/14 (100%)	9/10 (90%)	5/9 (56%)	0.152	0.127
**PTH **						
	**Baseline *(n =* 28), mean [95% CI]**	-	2.10 [1.26,3.52]	1.53 [0.94,2.49]		
	**Final *(n =* 22), mean [95% CI]**	-	1.63 [1.01,2.66]	2.49 [1.61,3.85]	-	0.011 ^5^
**Cord serum [25(OH)D] (*n =* 23)**						
	**Mean [95% CI]**	-	117 [99,137]	98 [84,115]	-	0.074
	**Range (min, max)**	-	74, 168	53, 124		
	**Cord [25(OH)D] ≥** ** 50 nmol/L, # */n* (%)**	-	12/12 (100%)	11/11 (100%)	-	1.000
	**Cord [25(OH)D] ≥** ** 80 nmol/L, *#/n* (%)**	-	11/12 (92%)	10/11 (91%)	-	0.949

^1^ Summary measures are geometric means with 95% confidence intervals, unless otherwise indicated. ^2^ Linear regression models (GEE was implemented where there were repeated measures for the same individuals) unless otherwise indicated; all *p* values < 0.05 remained significant after correction for multiple pairwise comparisons using the Holm method. ^3^ AUC for each group was the geometric mean (and 95% confidence intervals) of individual participants’ AUCs; the analyses included 33 participants who were followed-up to at least week 10 (day 63 or 65, depending on serum sampling schedule): NH, *n* = 14 participants; PH, *n* =10; PL, *n* = 9. Comparison of the AUC based on only 3 datapoints (baseline, day 21/28/35, and day 63/65) was undertaken as a sensitivity analysis because group PL participants only had [25(OH)D] measured at a maximum of three visits at which blood collection was scheduled; the latter analysis involved the same 33 participants as in the preceding analysis. ^4^ Proportion of participants in each group with average [25(OH)D] ≥ 50 nmol/L or ≥80 nmol/L in specimens collected on days 63 to 70; comparisons between groups were assessed by binomial regression. None of the pairwise comparisons were statistically significant after correction for multiplicity using the Holm method. ^5^* p* value for the group-by-time interaction term in a GEE model (exchangeable correlation and robust standard errors), using log-transformed PTH as the outcome, indicating that the change from baseline over time significantly differed between the two groups.

### 3.2. Effect of Prenatal Vitamin D3 Supplementation on Vitamin D Status

**Figure 3:**
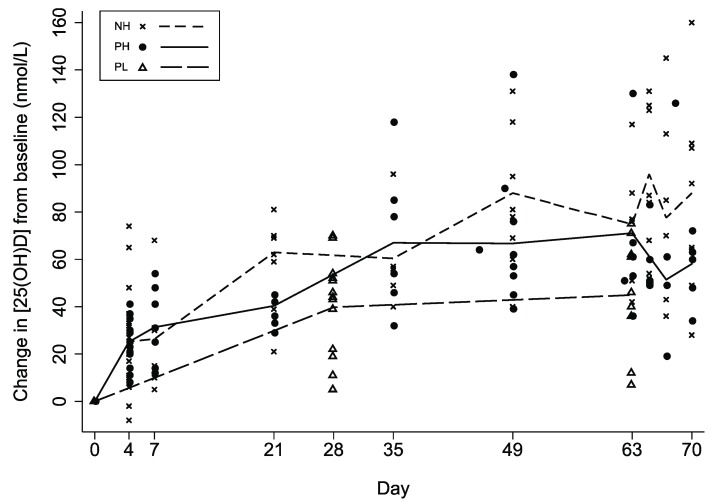
Changes in serum 25-hydroxyvitamin D concentration from baseline resulting from weekly vitamin D3 administration to non-pregnant women who received an initial dose of 70,000 IU and then 35,000 IU/week thereafter (NH), pregnant women who received an initial dose of 70,000 IU and then 35,000 IU/week thereafter (PH), and pregnant women who received 14,000 IU/week (PL). Lines connect the group means at each follow-up visit.

**Figure 4:**
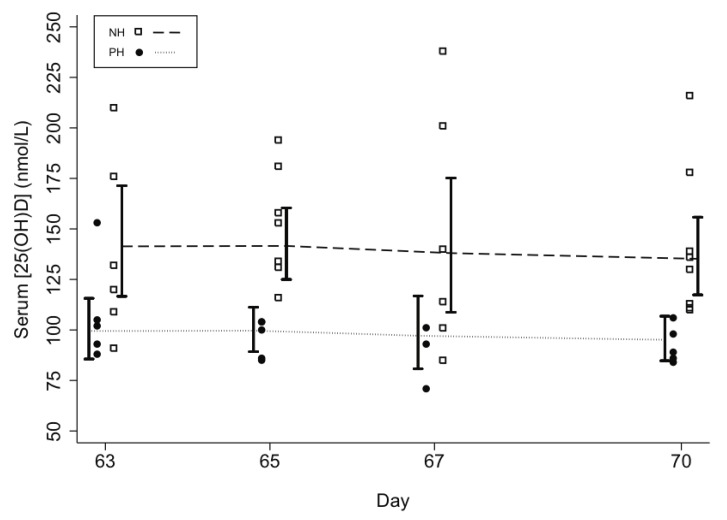
Lack of inter-dose fluctuations in mean serum 25-hydroxyvitamin D concentrations among non-pregnant (NH) and pregnant women (PH) during the 10th week of supplementation with 35,000 IU vitamin D3 per week, with the most recent dose administered on day 63. Lines connect the group means at each day; 95% confidence intervals are represented by vertical capped bars.

Mean [25(OH)D] rose gradually above baseline in all groups during follow-up (Table 2; Figure 3). Final mean [25(OH)D] during the 10th week of supplementation was significantly higher in PH *versus* PL (98 *vs.* 76 nmol/L, respectively; *p* = 0.038) and significantly lower *versus* NH (98 *vs.* 139 nmol/L; *p* < 0.001) (Table 2). However, ∆[25(OH)D] in PH was not significantly lower in the 10th week compared to NH (Table 2). The [25(OH)D] threshold of 50 nmol/L was attained by nearly all participants, but only the higher-dose regimen reliably led to [25(OH)D] ≥ 80 nmol/L by the 10th week in pregnant women. During the 10th week, there were no notable inter-dose fluctuations in NH and PH (Figure 4); mean [25(OH)D] at days 65, 67, and 70 differed from day 63 by <6 nmol/L (all *p* values > 0.5). There was substantial inter-subject variability in the response to vitamin D supplementation, with one PL participant demonstrating only a 7 nmol/L final increase in [25(OH)D] above her baseline. Among participants who received the higher-dose regimen, there was as much as a 7-fold difference between the lowest and highest responders based on ∆[25(OH)D] at week 10 (Table 2). Three participants in NH had [25(OH)D] > 200 nmol/L, but the highest [25(OH)D] in any pregnant participant was 153 nmol/L. There was no significant association between baseline vitamin D status and ∆[25(OH)D] (data not shown).

**Figure 5:**
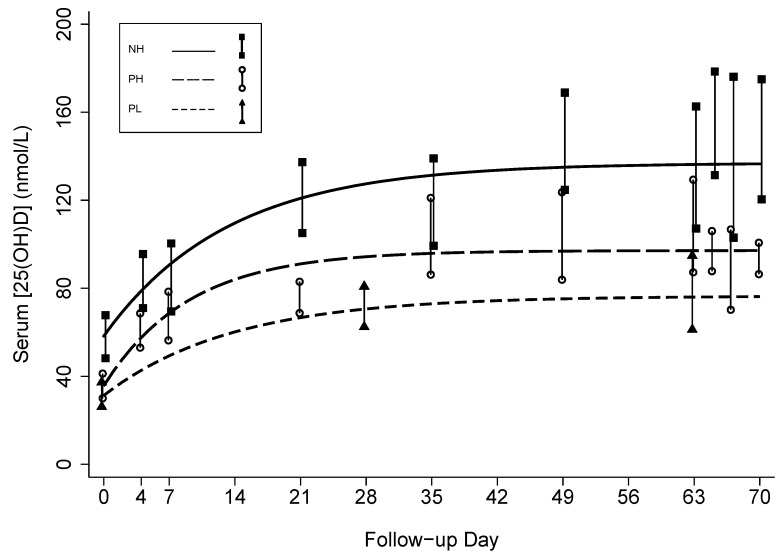
Negative exponential models predicting serum 25-hydroxyvitamin D concentrations in response to weekly vitamin D3 supplementation in non-pregnant women who received an initial dose of 70,000 IU and then 35,000 IU/week (NH), pregnant women who received an initial dose of 70,000 IU and then 35,000 IU/week (PH), and pregnant women who received a weekly dose of 14,000 IU/week (PL). Vertical bars represent the 95% confidence intervals of the empiric geometric means at each scheduled follow-up time.

Non-linear parametric models representing the change in [25(OH)D] as a continuous function of time yielded inferences regarding baseline and modeled steady-state mean [25(OH)D] that were consistent with the empiric cross-sectional estimates (Table 3) and provided appropriate fits to the data (Figure 5). Extended models indicated that the ∆[25(OH)D] at modeled steady-state was 19 nmol/L greater in PH compared to PL (*p* = 0.044) (Table 3). Mean modeled steady-state ∆[25(OH)D] was lower in PH compared to NH but the difference was not statistically significant (Table 3).

**Table 3:** Estimates of the change in serum 25-hydroxyvitamin D concentration over time in response to weekly vitamin D3 supplementation in non-pregnant women who received an initial dose of 70,000 IU and then 35,000 IU/week (NH), pregnant women who received an initial dose of 70,000 IU and then 35,000 IU/week (PH), and pregnant women who received a weekly dose of 14,000 IU/week (PL). Results are based on negative exponential models, and shown as mean (lower 95% confidence bound, upper 95% confidence bound).

		Model 1	Model 2	Model 3	Model 4	Model 5
		Non-pregnant (NH)	Pregnant, higher-dose (PH)	Pregnant, lower-dose (PL)	Pregnant (PL & PH)	Higher dose (NH & PH)
**Number of participants**		16	14	14	28	29
**Number of specimens**		89	75	36	111	162
**Baseline [25(OH)D] **	nmol/L	58 [48,69]	36 [29,42]	31 [25,38]	31 [25,37]	57 [47,67]
**∆[25(OH)D] at steady-state (*a*)**	nmol/L	79 [60,97]	62 [48,75]	45 [23,67]	43 [29,57]	77 [62,93]
**∆[25(OH)D] at steady-state per daily dose equivalent**	nmol/L/mcg D3 per day	0.63 [0.48, 0.78]	0.49 [0.38, 0.60]	0.90 [0.47,1.34]	-	-
**Steady-state [25(OH)D] ([25(OH)D]*_t_*_0_ + *a*)**	nmol/L	137 [116,157]	97 [87,108]	76 [54,98]	74 [61,87]	134 [117,151]
**Decay rate (*k*)**	days^−1^	0.08 [0.03,0.12]	0.11 [0.07,0.15]	0.07 [−0.01,0.16]	0.11 [0.07, 0.15]	0.09 [0.06, 0.12]
**Group (g)**	0 (Ref)	-	-	-	PW-C	NP-H
	1	-	-	-	PW-H	PW-H
**Difference in [25(OH)D] between groups at baseline (*β*)**	nmol/L	-	-	-	4 [−4,13]	−21 [−33,−9]
**Difference in ∆[25(OH)D] between groups at steady-state (*d*)**	nmol/L	-	-	-	19 [1,37]	−15 [−34,5]
					*p* = 0.044	*p* = 0.131
**Adjusted R^2^**		0.55	0.71	0.63	0.72	0.69

Mean cord serum [25(OH)D] was higher in PH (117 nmol/L) *versus* PL (98 nmol/L) but the difference was not significant (Table 2). The proportions of newborns with [25(OH)D] ≥ 80 nmol/L (PH: 92%; PL: 91%) and ≥50 nmol/L (PH: 100%; PL: 100%) were similar in the two groups. There was a moderate association between cord and maternal [25(OH)D] (ρ = 0.67, *p* < 0.001).

### 3.3. Ancillary Biochemical Parameters

**Figure 6:**
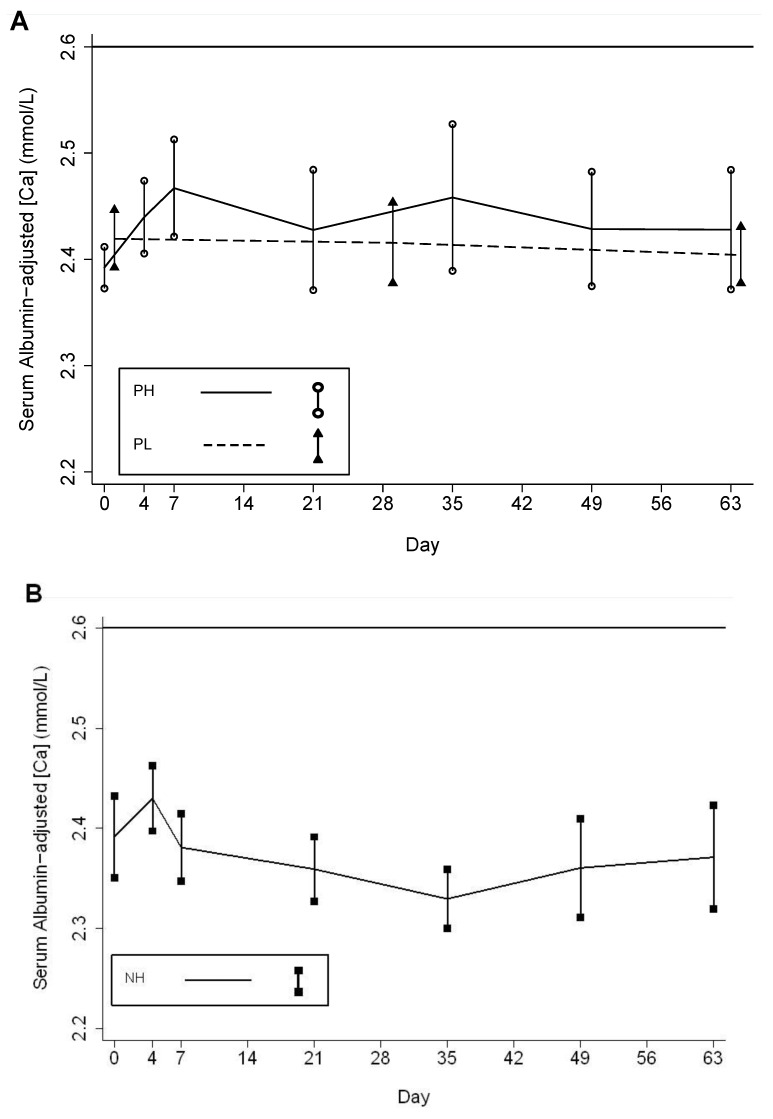
Mean albumin-adjusted serum calcium concentrations in the three participant groups. (**A**) Mean albumin-adjusted serum calcium concentration in pregnant participants who received an initial dose of 70,000 IU and then 35,000 IU/week (PH) and pregnant participants who received a weekly dose of 14,000 IU/week (PL); (**B**) Mean albumin-adjusted serum calcium concentration in non-pregnant participants who received an initial dose of 70,000 IU and then 35,000 IU/week (NH). Vertical bars represent the 95% confidence intervals of the means at each scheduled follow-up time. Horizontal line indicates the upper limit of the reference range (2.60 mmol/L).

Mean albumin-adjusted serum [Ca] increased significantly within the reference range during follow-up in PH but it did not change significantly in the comparison groups (Table 4; Figure 6). The increase in PH was significantly greater than in PL or NH (Table 4). There was a single episode of albumin-adjusted [Ca] > 2.60 mmol/L in a PH participant during an episode of acute gastroenteritis that occurred after two weeks of supplementation. Her albumin-adjusted [Ca] of 2.61 mmol/L declined to 2.39 mmol/L in a repeat specimen on the same day, the illness was self-limited, and there was no other biochemical or clinical evidence of vitamin D toxicity; furthermore, the participant continued to receive the supplement and had increasing [25(OH)D] (range, 52 to 98 nmol/L during follow-up) but did not develop any further episodes of hypercalcemia or elevations in urine ca:cr. There were no episodes of confirmed hypercalcemia according to *a priori* study definitions. 

**Table 4:** Albumin-adjusted serum calcium concentration at baseline, the 1st to 5th week of follow-up, and the 6th to 10th week of follow-up in non-pregnant women who received an initial dose of 70,000 IU vitamin D3 and 35,000 IU/week (NH), pregnant women who received an initial dose of 70,000 IU vitamin D3 and 35,000 IU/week (PH), and pregnant women who received 14,000 IU/week (PL).

	*n* ^1^			Albumin-adjusted serum calcium concentration (mmol/L)					# Episodes		
				Mean ± SD			*p* value,	*p* value,			
				Minimum, Maximum					>2.60 mmol/L		
							PH *vs.* NH ^2^	PH *vs.* PL ^2^			
Follow-up period	NH	PH	PL	NH	PH	PL			NH	PH	PL
**Baseline**	16	14	14	2.39 ± 0.08	2.39 ±0.04	2.42 ±0.05	-	-	0	0	0
				2.22, 2.5	2.3, 2.45	2.35, 2.52					
**1st to 5th week**	31	27	14	2.40 ± 0.07	2.45 ±0.07 ^3^	2.42 ±0.07	0.020	0.012	0	1 ^4^	0
				2.25, 2.6	2.32, 2.61	2.33, 2.55					
**6th to 10th week**	43	33	12	2.38 ± 0.07	2.44 ±0.07 ^5^	2.42 ±0.05	0.009	0.055	0	0	0
				2.2, 2.52	2.27, 2.57	2.33, 2.52					
**Total **	88	74	40	2.39 ± 0.07	2.43 ±0.07	2.42 ±0.06	0.991	0.104	0	1	0
				2.2, 2.6	2.27, 2.61	2.33, 2.55					
**Cord Serum**	-	12	11	-	2.69 ± 0.12	2.73 ± 0.13	-	0.414	0	0	0
					2.37, 2.82	2.56, 2.94					

^1^ Number of specimens (there may have been multiple specimens from a single participant during a given follow-up period). ^2^ Group-by-time interactions using GEE with robust standard errors. ^3^ Significant increase from baseline *p* < 0.001; remained significant after adjustment for multiple testing. ^4^ Isolated value of 2.61 mmol/L derived from uncorrected total serum calcium concentration of 2.67 mmol/L and serum albumin of 42.9 g/L. Repeat albumin-adjusted serum calcium later on the same day was 2.39 mmol/L (unadjusted [Ca] = 2.37 mmol/L). ^5^ Significant increase from baseline, *p* = 0.008; remained significant after adjustment for multiple testing. Not significantly different from 1st to 5th weeks, *p* = 0.654.

**Figure 7:**
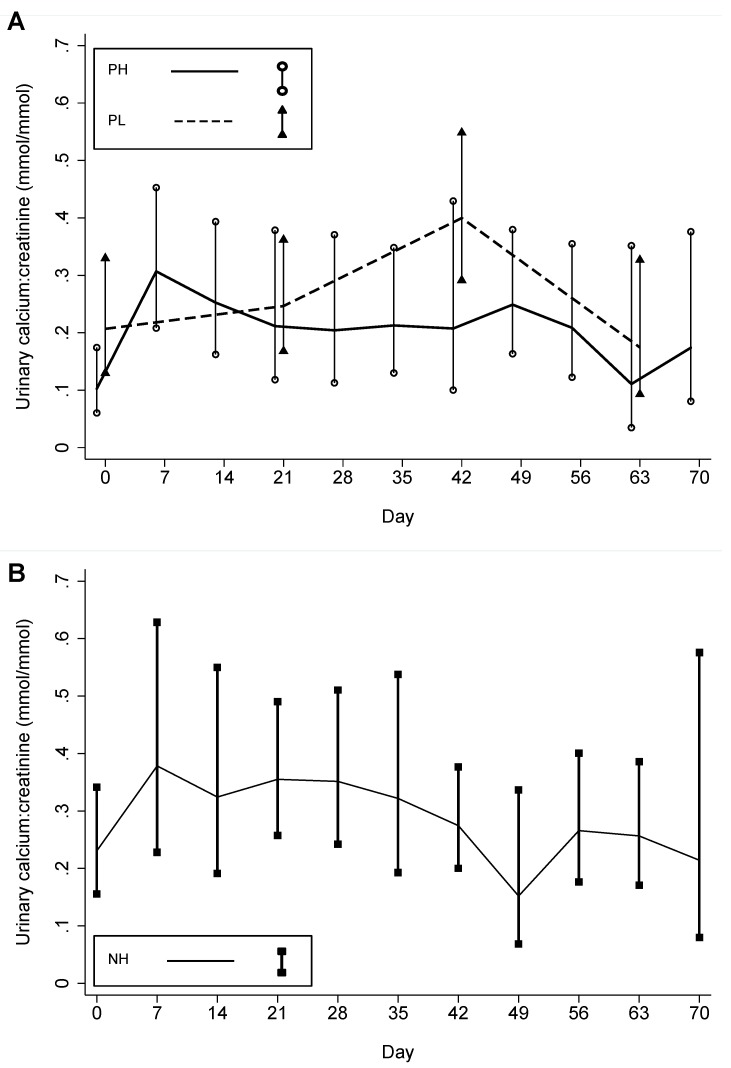
Mean urine calcium:creatinine ratio in the three participant groups.(**A**) Mean urine calcium:creatinine ratio in pregnant participants who received an initial dose of 70,000 IU and then 35,000 IU/week (PH) and pregnant participants who received a weekly dose of 14,000 IU/week (PL), and (**B**) Mean urine calcium:creatinine ratio in non-pregnant participants who received an initial dose of 70,000 IU and then 35,000 IU/week (NH). Vertical bars represent the 95% confidence intervals of the means at each scheduled follow-up time.

Urine ca:cr rose initially during follow-up in all groups but appeared to plateau in PL and decline in PH and NH during the latter half of the follow-up period (Table 5; Figure 7). There were five episodes of ca:cr > 1.0 mmol/mmol (Table 5). One participant in group PL had two consecutive episodes on days 42 and 44 and thus met the definition for persistent hypercalciuria by study criteria; however, [Ca] was normal and despite continued supplementation, the ca:cr was within the normal limits thereafter. The higher-dose intervention (PH) suppressed the average PTH concentration, which was significantly different from the increase observed in PL (*p* = 0.011) (Table 2).

**Table 5:** Urine calcium:creatinine ratio in random spot urine specimens collected at baseline, 1st to 5th weeks of follow-up, and 6th week to the end of the supplementation period in non-pregnant women who received an initial dose of 70,000 IU vitamin D3 and 35,000 IU/week (NH), pregnant women who received an initial dose of 70,000 IU vitamin D3 and 35,000 IU/week (PH), and pregnant women who received 14,000 IU/week (PL).

	***n*** **^1^**			**Urinary calcium-creatinine ratio (mmol/mmol)**					**# Episodes** **>1.0 mmol/mmol** (# Participants ever having >1.0 mmol/mmol)		
**Follow-up period**				**Mean** **^2^** **Minimum, Maximum**			***p* value** **PH *vs.* NH** **^3^**	***p* value** **PH *vs.* PL** **^3^**			
	**NH**	**PH**	**PL**	**NH**	**PH**	**PL**			**NH**	**PH**	**PL**
**Baseline**	16	14	14	0.23	0.10	0.21	-	-	0	0	0
				0.04, 0.58	0.01, 0.44	0.06, 0.91			(0)	(0)	(0)
**1st to 5th weeks**	49	36	12	0.36 ^4^	0.24 ^5^	0.24	0.164	0.105	3	0	0
				0.04, 1.47	0.02, 0.95	0.07, 0.64			(2)	(0)	(0)
**6th week to end **	62	53	33	0.26	0.18 ^6^	0.30	0.164	0.500	0	0	2
				0.03, 0.91	0.01, 0.96	0.05, 1.05			(0)	(0)	(1)
**Total **	127	103	59	0.29	0.19	0.26	0.014	0.047	3	0	2
				0.03, 1.47	0.01, 0.96	0.05, 1.05			(2)	(0)	(1)

^1^ Number of specimens (there may have been multiple specimens from a single participant during a given follow-up period). ^2^ Geometric means. ^3^ Group by time interactions using GEE with robust standard errors. ^4^ The *p* value for the test of the difference from baseline was 0.018; however, this was not statistically significant after adjustment for multiple testing (adjusted critical *p* value of 0.017). ^5^ The increase from baseline was statistically significant (*p* < 0.001) and remained so after adjustment for multiple testing (adjusted critical *p* value of 0.025). ^6^ Not significantly different from baseline after adjustment for multiple testing (*p* = 0.042, adjusted critical *p* of 0.025); and, not significantly different from the period of 1st to 5th weeks (*p* = 0.136).

### 3.4. Clinical Outcomes

There were no known supplement-related clinical adverse events. One pregnant participant in the lower-dose group (PL) developed new-onset mild hypertension unassociated with any significant morbidity or biochemical abnormalities (highest serum [25(OH)D] was 86 nmol/L); her pregnancy ended in an uncomplicated term delivery. The frequency of possible hypercalcemia symptoms was similar during follow-up in PH when compared to PL (odds ratio, 0.82; 95% CI, 0.35 to 1.92; *p* = 0.65). Groups PH and PL were similar with respect to pregnancy and newborn outcomes (Table 6). Anthropometric measures at birth did not significantly differ between the two groups (data not shown). 

**Table 6:** Pregnancy and newborn outcomes among women who received an initial dose of 70,000 IU vitamin D3 and 35,000 IU/week (PH) or 14,000 IU/week (PL) during the third trimester.

		PH	PL	*p* value (for between-group difference)
***n***		12	12	
**Gestational age at birth, weeks (by LMP)** **^1^**				
	**Mean (±SD)**	39.2 (±2.3)	38.5 (±2.7)	0.512
	**Range**	33.6–42.3	32.7–43.2	
**Preterm, # (%)**		1 (8%)	3 (25%)	0.590
**Birth weight (g)**				
	**Mean (±SD)** **^2^**	2774 (±456)	2604 (±379)	0.332
	**Range**	2210–4000	2020–3120	
	**# (%) SGA** **^3^**	9 (75%)	8 (67%)	1.000
	**# (%) LBW**	2 (17%)	4 (33%)	0.640
**Delivery mode, # (%) Cesarean section** **^4^**		6 (50%)	6 (50%)	1.000
**Sex, # (%) female**		6 (50%)	6 (50%)	1.000
**Live births** **^5^, # (%)**		12 (100%)	12 (100%)	-
**Alive at 1 month of age, # (%)**		12 (100%)	12 (100%)	-

^1^ In a sample of 113 deliveries at the study site (October 2009 to January 2010) for which there was a recalled first day of last menstrual period, the mean gestational age at birth was 39.7 weeks (±2.2). ^2^ In a consecutive sample of 362 liveborn infants delivered at the study site (October 2009 to January 2010), the mean birth weight was 2780 g (±440). ^3^ Based on US newborn birthweight reference [19]. ^4^ In a consecutive sample of 369 deliveries at the study site (October 2009 to January 2010), there were 199 cesarean deliveries (54%). ^5^ In a sample of 369 deliveries at the study site (Oct 2009 to Jan 2010), there were 7 stillbirths (2%).

## 4. Discussion

This study demonstrated the biochemical dose response to third-trimester high-dose weekly antenatal vitamin D3 supplementation. Among Bangladeshi women with a mean [25(OH)D] of 33 nmol/L, 70,000 IU followed by 35,000 IU/week of vitamin D3 until delivery yielded an average [25(OH)D] that was about 20 nmol/L higher than an antenatal dose of 14,000 IU/week (the IOM vitamin D upper limit at the time the study was conducted). Similar to our conclusions from analyses of single-dose vitamin D3 pharmacokinetics in the same study setting (and involving an overlapping group of participants) [13], we found that the minor differences between pregnant *vs.* non-pregnant participants receiving the same dose were within the margins of error given the small sample size. However, based on the present analysis, we could not exclude the possibility of a slightly diminished 25(OH)D response to a weekly dose of vitamin D during the third trimester of pregnancy. 

To our knowledge, the 35,000 IU/week regimen used in this study is the highest vitamin D3 maintenance dose studied in pregnancy under controlled conditions. Devlin *et al.* (1986) reported that a daily dose of 1000 IU vitamin D3 administered to 15 French women during the third trimester modestly raised mean maternal serum [25(OH)D] from 55 nmol/L to 65 nmol/L [20]. The largest published study of vitamin D3 supplementation in pregnancy was conducted by Bruce Hollis and colleagues in South Carolina, in which 502 pregnant women at 12 to 16 weeks gestation were randomized to 400 IU/day, 2000 IU/day, or 4000 IU/day vitamin D3 [21]. This population was more vitamin D-replete at baseline (mean [25(OH)D] = 60 nmol/L) compared to the present study. Based on data from the 350 participants (70%) followed until delivery, the 2000 IU/day and 4000 IU/day regimens raised [25(OH)D] to means of 105 nmol/L (rise of 47 nmol/L) and 119 nmol/L (rise of 60 nmol/L), respectively, at one month before delivery [21]. The Δ[25(OH)D] in the 2000 IU/day group in the Hollis study was similar to the response we observed in the 14,000 IU/day group (equivalent regimen) in the present study, substantiating the consistency of vitamin D3 dose-response modeling across diverse populations of pregnant women. In a separate trial in South Carolina, Wagner *et al.* reported comparatively less robust responses to 2000 IU/day and 4000 IU/day during pregnancy, which may have been attributable to non-adherence to the supplementation regimen [22].

The lower dose produced a more efficient 25(OH)D response per mcg of vitamin D3 when compared to the high-dose regimen: 0.73 *vs.* 0.46 nmol/L/mcg/day in the empiric estimates, and 0.90 *versus* 0.49 nmol/L/mcg/day based on the pharmacokinetic model. These estimates, as well as those from the non-pregnant cohort that received the higher-dose regimen (0.61 nmol/L/mcg/day based on 10th-week data, and 0.63 nmol/L/mcg/day based on the parametric model), were similar to the values conventionally cited for non-pregnant adults: ~0.70 nmol/L/mcg/day [23,24]. However, analyses by Barger-Lux *et al.* (1998) [25] and Aloia *et al.* (2008) [24], as well the recent IOM report (2010) [1], have demonstrated that the ∆[25(OH)D] per mcg is a curvilinear inverse function of vitamin D intake at doses <50 mcg/day, but nearly proportional to intake at >50 mcg/day [24], which may explain the greater observed efficiency of the lower dose.

A unique aspect of this study was the measurement of biochemical parameters *between* weekly doses at the end of the supplementation period. These data showed an absence of inter-dose perturbations in calcium homeostasis that might have otherwise been missed by sampling serum only at the time of the “trough” [25(OH)D] (*i.e.*, immediately preceding administration of a weekly dose). Although the study may have been too small to detect minor inter-dose fluctuations in [25(OH)D], the data supported the appropriateness of administering weekly doses of 35,000 IU instead of daily administration of 5000 IU.

In pregnant participants, the higher-dose vitamin D regimen had a significant suppressive effect on maternal PTH secretion, relative to the lower dose, as indicated by the change in average PTH concentrations from baseline to delivery, similar to previous observations by Wagner *et al.* in South Carolina [22]. However, since the role of PTH as a vitamin D status biomarker during pregnancy is unclear [26], the clinical significance of the apparent dose-response effect of vitamin D on PTH requires further study. 

Both the higher and lower vitamin D3 regimens administered to pregnant women attained fetal [25(OH)D] ≥ 50 nmol/L. Therefore, in this small sample, we did not observe a clear benefit of the higher-dose over the lower-dose regimen with respect to neonatal vitamin D status. In a related study at the same study site, we observed a mean cord [25(OH)D] of 50 nmol/L (range of 29 to 80 nmol/L) in a group of neonates born to women who had received a single vitamin D3 dose of 70,000 IU at 30 weeks gestation [13], and previous studies in South Asia have found cord serum [25(OH)D] ranging from 17 to 59 nmol/L [27,28,29,30]. 

Appreciable increases in serum calcium in the higher-dose relative to the lower-dose group highlighted a dose-dependent effect of vitamin D3 supplementation on calcium homeostasis. We previously reported that mean serum calcium concentrations rose slightly but significantly during the first week after administration of a single 70,000 IU dose of vitamin D3 in both pregnant and non-pregnant participant groups [13]. However, in the present analyses of weekly-dose vitamin D3, a significant increase in serum [Ca] from baseline was only observed in pregnant women who received the higher dose. Pregnancy is associated with an elevation in the maternal serum concentration of the active vitamin D metabolite, 1,25-dihydroxyvitamin D (1,25(OH)2D) [31,32], which appears to be primarily attributable to classic renal 1α-hydroxylation of 25(OH)D [33]. However, placental trophoblasts and decidual cells [34] are capable of extra-renal 1α-hydroxylation which could theoretically predispose the pregnant woman to exaggerated physiological responses to increases in [25(OH)D] [9]. Similar to the participants who received only a single dose of 70,000 IU [13], maternal serum calcium values in the weekly-dose participants were all below the threshold for defining hypercalcemia used by the IOM in setting the 1997 dietary reference intakes (DRIs) for vitamin D (2.75 mmol/L) [35] and in the revised DRIs in 2010 (2.63 mmol/L) [1]. Cord blood calcium concentrations were also within reference limits, and [25(OH)D] were well below the range that has been associated with toxicity in adults [36] and older children [37]. Pregnancy and newborn clinical outcomes were within the expected range for the study population, but we were unable to draw conclusions from this study regarding clinical effects of vitamin D. Nonetheless, this study together with the recent findings of Hollis and Wagner and colleagues in South Carolina [21,22] demonstrate that vitamin D3 doses during pregnancy up to 25% above the current IOM UL of 4000 IU/day do not induce hypercalcemia, and have not led to any observed short-term clinical adverse effects.

There were several important limitations of this study. First, precision of estimates of pharmacokinetic parameters and between-group comparisons, as well as the generalizability of inferences regarding maternal-fetal safety of high-dose vitamin D supplementation, were limited by the small number of participants, stringent inclusion/exclusion criteria, and enrolment of pregnant and non-pregnant participants at one clinic site. Moreover, the lower-dose pregnancy group had less frequent blood sampling (a cost-savings measure given the relative lack of safety concerns for this group) and only 9 of 14 enrolled women contributed endpoint samples during the 10th week of supplementation. The supplementation period may not have been long enough to ensure that all participants reached a steady-state [25(OH)D]. Conclusions based on comparisons between pregnant and non-pregnant women were tempered by the differences in baseline characteristics, including season of enrolment and the relatively higher socioeconomic status of the non-pregnant participants. In addition, there were too few participants to consider modifiers of Δ[25(OH)D]. Most importantly, the present results do not yet provide sufficient evidence that the regimens studied are beneficial or safe for use in clinical or public health practice; rather, they serve to inform application of these dose regimens in future research studies.

## 5. Conclusions

This detailed analysis of the response to high-dose weekly vitamin D3 administered during the third-trimester of pregnancy demonstrated a dose-responsiveness to oral vitamin D3 in Bangladeshi women that echoed observations in other settings, and was generally in accordance with established pharmacokinetic characteristics of vitamin D3. Nonetheless, increases in the mean calcium concentration (within the normal range) and suppression of PTH secretion among pregnant women receiving the higher-dose regimen (70,000 IU initial dose followed by weekly doses of 35,000 IU) highlighted the physiological impact of the intervention and the need to cautiously address potential pregnancy-specific sensitivities to vitamin D supplementation. 

Prior to undertaking large trials to test the effects of prenatal micronutrient interventions on pregnancy and birth outcomes, preliminary dose-finding and safety studies are essential, particularly when the intervention is a fat-soluble vitamin at a dose above the conventional upper limit of tolerability (*i.e.*, 4000 IU/day for vitamin D, as established by the Institute of Medicine [1]). The most direct application of the present observations is to guide the design of future trials of vitamin D3 (at doses up to 35,000 IU per week) aimed at confirming safety and establishing the health benefits of antenatal vitamin D supplementation in South Asia, where many potentially vitamin D-responsive outcomes (e.g., infant growth and infectious disease morbidity) are major public health priorities. Following from our preliminary pharmacokinetic studies, we have conducted a placebo-controlled trial of 35,000 IU/ week during the third trimester (*n* = 160), with follow-up of infants to monitor growth to one year of age (NCT01126528). Future trials in Dhaka will address the dose-dependency of the effects of prenatal vitamin D supplementation on infant growth and morbidity.

## Appendix

### Non-Linear Modeling of Change in *25[(OH)D]* over Time

Mean changes in [25(OH)D] over time in each group were modeled as continuous non-linear parametric functions. Consistent with the first-order process that characterizes 25(OH)D metabolism in the physiological range of vitamin D inputs, Heaney’s group has shown empirically that a negative exponential growth function is well suited to model the gradual rise in [25(OH)D] over time (*t*) to a steady-state plateau in response to daily (Heaney *et al.*, 2003 [23]) or weekly (Heaney *et al.*, 2011 [38]) oral vitamin D3 supplementation:

(1)

A particular advantage of this model is that despite its non-linearity, the coefficients are easily interpreted: *a* is the Δ[25(OH)D] above baseline at steady-state and *k* is the slope that defines the rate of the rise (the higher is *k*, the more rapidly the steady-state is reached). The steady-steady is the [25(OH)D] at which the rate of 25(OH)D formation theoretically equals the rate of 25(OH)D utilization/catabolism. Furthermore, the model could be readily extended to permit comparisons between groups of participants (g), with the aim of estimating the average difference (d) between the groups’ [25(OH)D] at steady-state (see below for derivation of the extended model):

(2)

Regression coefficients were estimated by a non-linear least-squares approach, assuming a log-normal error distribution of [25(OH)D] and standard error estimation that accounted for the intra-subject correlation of repeated measures.

To derive an extended negative exponential growth function that enabled comparison of the steady-state concentrations between the two groups, we first considered a generic model for the negative exponential growth function, where [25(OH)D] at time *t* is a function of the baseline concentration [25(OH)D] at t=0, the slope of the exponential rise, *k*, and the asymptotic maximal rise above baseline, *a*, at steady-state (*t* = infinity):

We can consider Equation (1) with respect to two different groups, *g*, such that if:

Then,

(3)

Similarly for group *g* = 1, if:

Then,

(4)

Combining Equation (3) and Equation (4) into one model, and allowing the intercept to vary by group:

(5)

Solving further:

(6)

Since we are specifically interested in measuring the difference between *a*_1_ and *a*_0_, we can invoke a new coefficient *d*, whereby:

(7)

Then, substituting Equation (7) into Equation (6) yields:
